# Comparison of three different presbyopia-correcting intraocular lenses

**DOI:** 10.22336/rjo.2020.58

**Published:** 2020

**Authors:** Valerii Serdiuk, Svetlana Ustymenko, Svetlana Fokina, Ivan Ivantsov

**Affiliations:** * Dnipropetrovsk Regional Clinical Ophthalmological Hospital, Ukraine

**Keywords:** multifocal IOL, quality of vision, dysphotopsia, comparative, cataract

## Abstract

**Objective (aim):** to test the refractive and visual outcomes and the quality of vision after the bilateral implantation of three different multifocal intraocular lenses (MIOLs) in patients with age-related cataract.

**Methods:** In this retrospective, comparative study including 90 eyes of 45 cataract patients, bilateral implantation of either the hydrophilic trifocal Liberty® 677MY capsular bag IOL, the hydrophilic AT LISA® tri 839M lens, or the hydrophobic AcrySof® IQ PanOptix® IOL was performed during routine cataract surgery. Refractive outcomes, visual acuities (VA) for far, intermediate and near distances, as well as visual quality, dysphotopic events and spectacle use were evaluated six months postoperatively.

**Results:** VA curves were similar for the three MIOLs, however the Liberty lens seemed to be superior for far and near, while AT LISA tri provided somewhat better VA in the intermediate range. Refractive correction was the most effective with the Liberty IOL (p=0.0131). Dysphotopic phenomena were usually perceived in low light conditions. Their frequency was lower with the AT LISA tri and Liberty lenses. Symptoms were significantly less disturbing for patients implanted with the Liberty lens, two-thirds of AT LISA tri and Liberty patients, while only 57% of PanOptix patients achieved spectacle independence.

**Conclusions:** All examined MIOLs were found to be safe and efficient in presbyopia-correction of cataract patients, however different models had different advantages. The vision preferences of each patient should always be taken into consideration when choosing a MIOL, and the possible occurrence of dysphotopic events should be also clearly communicated in each case.

**Abbreviations:** ACD = Anterior chamber depth, ANOVA = Analysis of variance, AXL = Axial length, CDVA = Corrected distance visual acuity, CYL = Cylinder; Cylindric refraction, D = Diopter, IOL = Intraocular lens, K1; K2 = Keratometry values, MIOL = Multifocal intraocular lens, n = Number of cases, n.a. = Not applicable, Postop = Postoperative, QoV = Quality of Vision, SD = Standard deviation, SEQ = Spherical equivalent, SPH = Sphere; Spherical refraction, UDVA = Uncorrected distance visual acuity, UIVA = Uncorrected intermediate visual acuity, UNVA = Uncorrected near visual acuity, VA = Visual acuity

## Introduction

Although several presbyopia-correcting (multifocal) intraocular lenses (MIOLs) have been introduced on the market in the last two decades, the overall percentage of MIOL-implantations considering all cataract surgeries and clear lens extractions is of a moderate 7% [**1**].

Besides health insurance and other financial issues, the most dissuasive force behind doctors’ reluctance might be the frequent dissatisfaction of the patients [**1**]. De Vries et al. reported that the most common causes behind patients’ reduced satisfaction are usually residual ametropia (caused by untreated astigmatism, inappropriate IOL-power choice or refractive surprises due to higher order aberrations of the cornea) and insufficient visual acuity at one or more distances [**2**,**3**]. Usually, near vision is affected, hence the patient still requires spectacles for near vision tasks, and the initial aim of becoming spectacle independent fails to be achieved [**4**,**5**]. Although several studies report a high degree of patient satisfaction, many patients still complain about reduced contrast sensitivity, difficulties in mesopic and scotopic light conditions [**2**,**3**,**5**]. Furthermore, compared to monofocal IOLs, a major percentage of the patients is bothered by dysphotopic phenomena like haloes and glare or negative dysphotopsia [**1**-**4**,**6**,**7**].

The majority of MIOLs currently on the market are based on diffractive optical principles. These IOL surfaces are designed and manufactured so that the path of light rays entering the eye (or the surface of the lens) will be modified in a carefully designed, predetermined way, creating multiple focal points, and distributing light energy among these foci as desired [**3**,**8**]. Nevertheless, light cannot be allocated to one single focal point exclusively: in case of a larger pupil or low light conditions, most of the light energy is distributed into the focal point responsible for far vision, but a smaller amount of light still arrives at the intermediate and near foci [**8**,**9**]. Consequently, undesirable light phenomena might be experienced, which often bother the patients and reduce their quality of vision [**2**-**4**,**6**,**7**,**9**]. Additionally, the more the light energy is sent to the untargeted focal point, or is out-of-focus as a consequence of light scattering on each diffractive surface elements, the less sharp and detailed image the patient receives from the targeted focus, and contrast sensitivity decreases. These dysphotopic artifacts and adverse side effects belong to the main reasons of MIOL explantation [**2**-**4**,**6**,**7**]. 

The aim of our current investigation was to assess visual and refractive outcomes, and above all, the quality of vision with special attention paid to the occurrence of dysphotopic phenomena and patients’ satisfaction after the binocular implantation of one of three presbyopia-correcting MIOLs on the market: two market leaders, the AT LISA tri and the PanOptix lenses, and the Liberty trifocal IOL of an emerging manufacturer.

## Materials and Methods

**Subjects**

Forty-five patients binocularly implanted with one of the three investigational MIOLs were enrolled into our retrospective, single-center, single surgeon clinical investigation. As a retrospective study, no ethical approval was required in accordance with the applicable provisions of our country, however we received the approval of the National Ethical Committee during the data evaluation period of our study. The investigation was conducted in compliance with the tenets of the Declaration of Helsinki [**10**], and all sensitive patient data were handled with care during the whole data collection, processing and evaluation period. A written consent was obtained from each patient on contributing to the management of their pre- and postoperative data.

Only patients meeting the inclusion criteria were selected for the investigation. Special attention was paid so as not to include any patients who had been previously diagnosed with any pathology of the anterior or posterior segment of the eye (including corneal dystrophy, high degree of ametropia, pseudoexfoliation syndrome, macular disorders, diabetic retinopathy, etc.). Patients with any pathologies of the ligamentous apparat of the lens (subluxation) were omitted to the evaluations. Dry eye syndrome was a relative contraindication: in cases in which the dry-eye condition could be treated with copper-stone compensation, we decided to enroll the patients into the data analysis.

***Measured parameters***

Preoperative ocular examinations followed the routine protocol of our clinic in all cases. A thorough examination of both the anterior and posterior segments of each eye (Oculus Pentacam HR Scheimpflug camera by Oculus Optikgeräte GmbH; Wetzlar, Germany and Optovue RTVue 100-2 optical coherence tomograph by Optovue Inc; Fremont, CA, USA) was extended with the measurement of intraocular pressure (mmHg) using the HNT-7000 non-contact tonometer (Huvitz Corp.; Gyeonggi-do, Republic of Korea). For biometry measurements (axial length, AXL; keratometry 1 and 2 values, K1 and K2; anterior chamber depth, ACD) we used the IOLMaster 500 optical biometer (Carl Zeiss Meditec AG; Jena, Germany). Each parameter was registered in millimeters (mm). Based on the biometry measurements, the appropriate IOL-power for each eye was calculated with the SRK/ T formula in case of the Liberty and the AT LISA tri lenses, and with the Barrett formula in case of the PanOptix lens. The target refraction was emmetropia in all cases. All calculations were verified with the Z Calc online IOL calculator (Carl Zeiss Meditec AG; Jena, Germany). Pachymetry was performed using the Humphrey 740i Visual Field Analyzer (Carl Zeiss Meditec AG; Jena, Germany), and endothelial cell density (cells/ mm2) was determined with the EM-3000 specular microscope (Tomey Corp.; Nishi-ku Nagoya, Japan).

Refractive errors (sphere, SPH; cylinder, CYL) were determined by the Huvitz HRK-700 autorefractometer supplemented with Huvitz HDR-7000 automated phoropter (Huvitz Corp.; Gyeonggi-do, Republic of Korea), and also based on the subjective perception of each patient.

Monocular uncorrected and corrected distance visual acuities (UDVA, CDVA, respectively) at 5 meters, uncorrected intermediate visual acuities (UIVA) at 80 and 60 cm, and uncorrected near visual acuities (UNVA) at 40 and 30 cm were measured using the Cyrillic visual acuity chart set developed by the Moscow Scientific Research Institute of Ophthalmic Disease (Helmholtz) (Moscow, Russian Federation), and expressed on the decimal scale. Measurements were performed in photopic conditions (250-300 lumen/ mm2) in all cases.

All measurements were repeated shortly after the surgery and the implantation of the IOLs (within two months), then once between the second and sixth postoperative months, and finally after six months.

Postoperative evaluations were performed by the same person (II) as preoperatively.

***Surgery***

Cataract surgeries were performed by the same experienced surgeon (VMS) between April, 2017 and March, 2019, according to the same surgical protocol regardless of the implanted IOL model. Each patient was implanted with the same model in their both eyes. An average interval of one week was taken between the surgery of the first and the fellow eye in each case. The surgical procedure was a conventional phacoemulsification method. A clear corneal incision of 1.80 mm was used in case of the AT LISA tri and Liberty lenses, while a 2.0-2.2 mm cut was required in case of the PanOptix IOL. Either the AJL CELL 2% (AJL Ophthalmic, S.A.; Miñano, Spain) or the Alcon Viscoat (Alcon Inc.; Fort Worth, TX, USA) viscoelastic material was used during the surgeries. The AT LISA tri and Liberty Intraocular lenses were implanted using the Viscoject Bio 1.8 Injector Set (Carl Zeiss Meditec AG; Jena, Germany), while the Monarch III D Cartridges (Alcon Inc.; Fort Worth, TX, USA) were used in case of the PanOptix IOL. All wounds were left sutureless. Levofloxacin 0.5% eye drops were administered for two days before the surgery (four times a day), and six times a day during the first postoperative day. Then, four times a day for one week. Postoperative treatment was supplemented with 0.1% dexamethasone during the first postoperative month in each case to reduce the risk of inflammation and infection.

***Implanted MIOLs***

Three multifocal intraocular lenses widely used on the market were chosen for comparison. The main characteristics of the examined MIOL-models are summarized in **Table 1**. All investigated lenses use different approaches to ensure trifocal vision, which might have an impact on the quality of vision provided, and on the frequency, intensity and disturbing nature of dysphotopic side effects. The decision regarding which model to implant in a patient was based on the type of insurance and the financial means of the particular patient in each case. We also tried to take into account the personal preferences and/ or occupation of each patient (e.g. if they frequently drove at night), and their psycho-emotional profile (ability of neuroadaptation, and how likely they were to tolerate dysphotopic sensations). Our final IOL-choice was also greatly influenced by the IOL-calculation, and we preferred the IOL, which had given a predicted residual refraction the closest to emmetropia. Unfortunately, we did not have the opportunity to implant astigmatism-correcting toric IOLs, as we neither aimed to further divide our set of patients, nor had our patients had the appropriate budget to bear the costs of a toric lens. Therefore, this current work is limited to the examination of the non-toric models.

**Table 1:** Characteristics of the three examined trifocal intraocular lenses

Characteristic	AT LISA tri 839MP	Liberty 677MY	AcrySof IQ PanOptix TFNT00
Optic material	Hydrophilic acrylic (25 %) with hydrophobic surface properties	Hydrophilic-hydrophobic acrylic copolymer	Hydrophobic acrylic
Refractive index	1.46	1.46	1.55
Abbe number	58	58	37
Optic design	Aspheric, diffractive, Smooth Micro Phase technology	Biconvex, 360° Special Square Edge*, anterior and posterior aspheric surface	Biconvex, square edges, anterior aspheric surface
Diffractive surface	Anterior	Anterior, 3.0 mm	Anterior surface, 4.5 mm
Light loss	14.3% (average)	11%	12%
Light energy split with 3.0 mm pupil	50% D / 20% I / 30% N	53% D / 14% I / 33% N	50% D / 25% I / 25% N
Optic diameter (mm)	6.0	6.0	6.0
Length (mm)	11.0	13.0	13.0
Haptic configuration	Plate haptics	Double C-loop	Modified L
Haptic angulation (°)	0°	0° with posterior vaulting	0°
Ultraviolet filter	Yes	Yes + blue light filter	Yes + blue light filter
Diffractive steps	21 to 29	7	15
Addition	+1.66 dpt and 3.33 dpt	+1.75 dpt and 3.50 dpt	+2.17 dpt and +3.25 dpt
A-constant (SRK/T)	118.6	118.9	119.1

**AT LISA tri 839MP**

The optic surface of the AT LISA tri family consists of a trifocal center (4.34 mm in diameter) and bifocal periphery (from 4.34 to 6.0 mm in diameter). The diffractive elements of the AT LISA tri optic do not have any sharp angles, but are designed with smooth transitions between each diffractive step. According to the manufacturer, this results in high optical image quality with reduced light scattering. The light is distributed asymmetrically between the near and distant focal points, and so it is supposed to improve intermediate vision and to greatly reduce halos and glare.

**Liberty 677MY**

The anterior diffractive optic of the Liberty IOL family consists of seven concentric apodised diffractive steps which take 25% of the IOL surface (within a 3.00 mm diameter), leaving 75% of the lens surface purely refractive. As considerable additional light scattering is usually caused by the imperfections of manufacturing and above all by the number of the diffractive steps, Medicontur has limited the number of diffractive rings to seven, which is remarkably less compared to other trifocal lenses on the market. Liberty IOLs are strongly pupil dependent using the near triad reflex, which implies miosis under accommodation. Light distribution among the three focal points follows natural ocular physiology, shifting light energy allocation towards the distance focus as the pupil dilates, and in parallel, letting less light energy into the near focus in order to reduce dysphotopsia.

**PanOptix TFNT00**

The posterior lens surface of the PanOptix lens is spherical, and the anterior surface is aspheric with a diffractive surface on the central 4.5 mm portion of the optic zone. The lens is based on a quadrifocal design and uses a proprietary optical technology to redistribute the focal point at 120 cm to the distance focal point for amplified performance. Light is split to three foci (distance, intermediate at 60 cm, and near at 40 cm). The optical surface is reported to transmit 88% of the light to the retina at a 3.0 mm pupil size, and provides optimized performance in a wide range of lighting conditions due to low dependence on the pupil size.

***Dysphotopsia – Quality of vision evaluation***

According to the method described by McAlinden et al., dysphotopsia was tested for six months postoperatively [**11**]. Briefly, a 30-item instrument was designed with 10 symptoms (glare, halo, starburst, blurry vision, etc.) rated in each of three scales (frequency, severity, and bothersome). For the first seven quality of vision (QoV) symptoms, an accompanying image published along with the description of the method was applied to help patients understand the questions and to reduce the possibility of inconsistent responses [**11**]. Responses were scored on a scale from 0 to 3. Zero represented the most, while 3 represented the least favorable outcome in each case. Nevertheless, the questionnaire itself labels each response category with descriptive wording to help the patients choose the most appropriate rating. Questionnaires were filled by each patient by themselves. 

The response distribution of each question was investigated and plotted in the three study groups defined by the three MIOLs. Furthermore, the mean and standard deviation (SD) of responses given to each question were also calculated, and the results of the three lenses were compared.

***Statistical analysis***

Pre- and postoperative data of 90 eyes (45 patients) were collected in Microsoft Excel (Microsoft Inc.; Redmond, WA, USA), and analyzed using the GraphPad Prism 8.4.0 software (GraphPad Software Inc.; San Diego, CA, USA). Descriptive statistics (mean; standard deviation, SD; minimum and maximum values; median; 95% confidence intervals) were calculated for all variables. Normality test was performed according to the D’Agostino & Pearson method in each case, and further test for comparing two or more variables were chosen based on the results. Comparisons between matching variables were performed using either the two-tailed t-test (in the case of normal distribution) or the Wilcoxon matched-pairs signed rank test (when a non-parametric test was required). Comparison of more than two groups was performed using the non-parametric type of the one-way analysis of variance (ANOVA; Kruskal-Wallis test) followed by Dunn’s multiple comparison test. Visual acuity curves of the three groups defined by the implanted IOL were compared with the two-way ANOVA test followed by Sidak’s multiple comparisons test.

Results were presented as mean ± standard deviation (SD) along with the range defined by the minimum and maximum values in the case of each variable. P values of 0.05 or less were considered to be statistically significant in all cases.

## Results

Pre- and postoperative data of 90 eyes (45 patients) were included in the analysis. The collection of all analyzed data is available upon well-justified requests sent to the correspondent author. Fourteen patients (28 eyes) received the AT LISA tri IOL, 17 patients (34 eyes) were implanted with the Liberty, and 14 patients (28 eyes) with the PanOptix lens. The average age of the population was 57.6 ± 14.9 years, although the subgroup of patients implanted with the PanOptix lens were younger than those in the other two subgroups (p=0.0059). Detailed demographic characteristics, biometry values, mean preoperative refractive errors and UDVA and CDVA measured in the three groups are presented in **Table 2**.

**Table 2:** Demographics and preoperative characteristics of the three subsets of patients

Demographic	AT LISA tri 839MP	Liberty 677MY	PanOptix TFNT00	Significance (p)
Number of eyes (patients)	28 (14)	34 (17)	28 (14)	
Gender (patients; n; %)				
Female	6 (42.9%)	11 (64.7%)	7 (50.0%)	
Male	8 (57.1%)	6 (35.3%)	7 (50.0%)	0.4577
Age (years)				
Mean ± SD	55.2 ± 14.6	65.9 ± 13.7	49.7 ± 12.3	
Range	31; 78	34; 83	35; 71	0.0059*
Axial length (mm)				
Mean ± SD	23.73 ± 0.63	23.85 ± 0.64	23.60 ± 0.54	
Range	22.81; 24.62	22.74; 24.98	22.69; 24.80	0.2847
K1 (mm)				
Mean ± SD	7.91 ± 0.30	7.88 ± 0.26	7.77 ± 0.24	
Range	7.16; 8.62	7.39; 8.55	7.14; 8.11	0.1587
K2 (mm)				
Mean ± SD	7.85 ± 0.21	7.78 ± 0.21	7.60 ± 0.28	
Range	7.41; 8.33	7.44; 8.29	6.80; 7.95	0.0007*
IOL-power (D)				
Mean ± SD	19.7 ± 2.80	19.8 ± 3.07	21.1 ± 1.87	
Range	15.5; 24.5	14.0; 25.5	17.0; 24.5	0.1012
SPH preoperative (D)				
Mean ± SD	-0.01 ± 2.35	0.08 ± 2.12	0.96 ± 1.77	
Range	-6.25; +4.50	-4.50; +5.00	-2.25; +4.25	0.1707
CYL preoperative (D)				
Mean ± SD	-0.52 ± 0.63	-0.83 ± 0.63	-0.77 ± 0.47	
Range	-1.75; +1.00	-2.50; +0.50	-2.00; +0.25	0.1979
UDVA (decimal)				
Mean ± SD	0.32 ± 0.27	0.33 ± 0.23	0.33 ± 0.29	
Range	0.01; 0.90	0.01; 0.90	0.02; 0.80	0.8698
**Notes:** * p values of 0.05 or less were considered to be statistically significant in all cases.				

***Visual acuity***

The average preoperative uncorrected distance visual acuities were similar in the three studied groups (p=0.8698; **Table 2**).

**Fig. 1:**
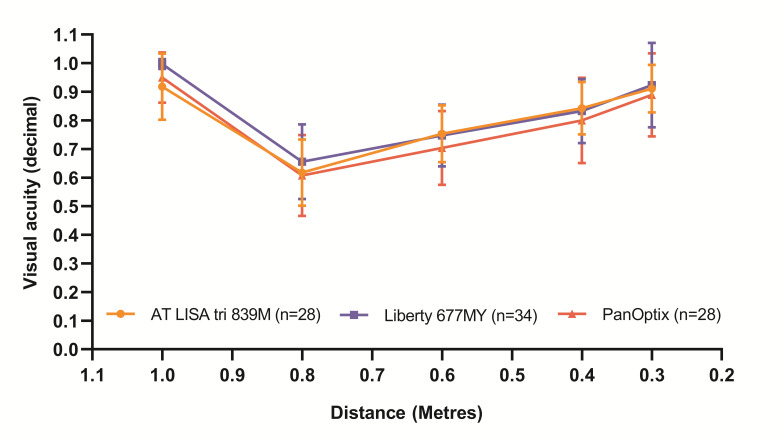
Monocular uncorrected visual acuity curves are similar in case of the three trifocal intraocular lenses. Data points are plotted as mean ± standard deviation

During the six-month postoperative visit, monocular uncorrected visual acuity curves were plotted from VAs measured at 5 meters, 80, 60, 40 and 30 cm for each group defined by the implanted MIOLs. The three curves were similar (**Fig. 1**; p=0.1433), however, a significant difference between the AT LISA tri and the Liberty group could be found at 5 meters (AT LISA tri UDVA= 0.92 ± 0.12; Liberty UDVA=1.00 ± 0.02; p=0.0232).

**Fig. 2:**
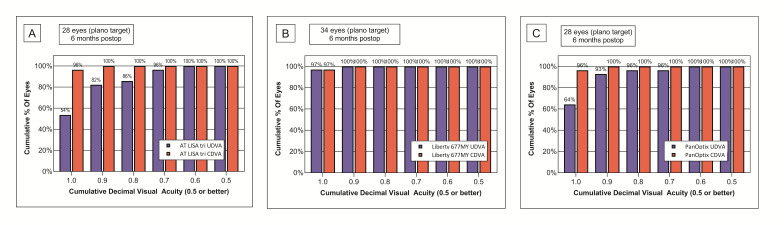
Monocular uncorrected and corrected visual acuities measured six months postoperatively show that the Liberty 677MY IOL was the most efficient in restoring distance vision. (A) AT LISA tri 839MP; (B) Liberty 677MY; (C) PanOptix TFNT00

A more detailed analysis of the cumulative decimal uncorrected visual acuities at each distance revealed that the best distance visual acuities could be achieved with the Liberty lens (**Fig. 2**), and this was the group in which the UDVA values were the closest to the CDVA values.

**Fig. 3:**
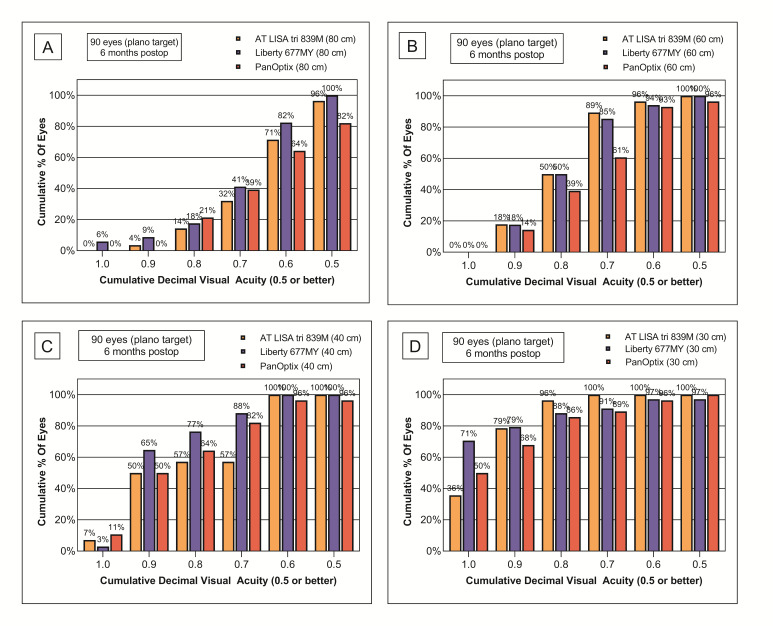
Uncorrected intermediate and near visual outcomes measured at 80, 60, 40 and 30 cm show that both AT LISA tri 839MP and Liberty 677MY are superior in restoring intermediate and near vision, compared to the PanOptix lens. (A) Intermediate visual acuity measured at 80 cm; (B) Intermediate visual acuity measured at 60 cm. (C) Near visual acuity measured at 40 cm; (D) Near visual acuity measured at 30 cm

At the intermediate 80 cm reading distance, the Liberty IOL was superior to the other two lenses, however, at 60 cm the AT LISA tri lens provided the best intermediate visual outcomes. The UIVA results achieved with the PanOptix IOL were inferior to the other two IOLs throughout the whole intermediate range (**Fig. 3a, b**).

Monocular uncorrected near visual acuities (UNVA) showed similar results reflecting the advantage of the AT LISA tri and Liberty lenses over the PanOptix model. The AT LISA tri had better results at a 30 cm reading distance compared to the VAs measured at 40 cm, while the Liberty provided similarly good near vision at both distances (**Fig. 3c, d**).

***Correction of refractive errors***

**Fig. 4:**
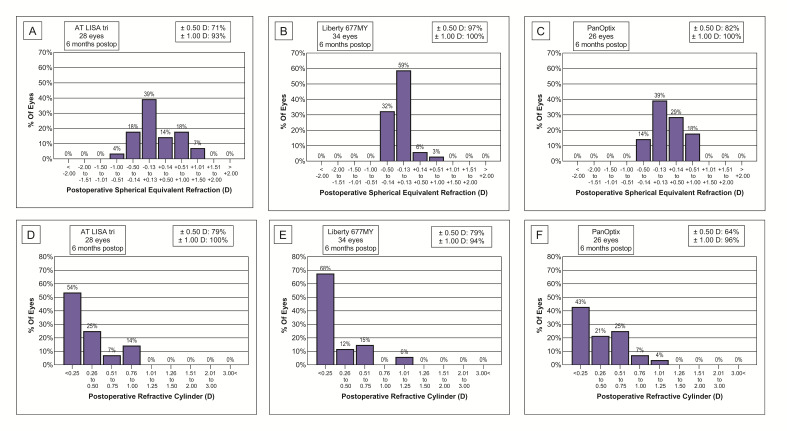
Postoperative residual spherical equivalent refractions and cylindric errors prove the efficiency of the three multifocal intraocular lenses in correcting refractive errors. Liberty 677MY has brought the most favorable outcomes, however, astigmatism might have reduced the results in case of the PanOptix lens. (A) Residual SEQ with the AT LISA tri 839MP IOL; (B) Residual SEQ with the AT LISA tri 839MP IOL; (C) Residual SEQ with the PanOptix TFNT00 IOL; (D) Residual CYL with the AT LISA tri 839MP IOL; (E) Residual CYL with the AT LISA tri 839MP IOL; (F) Residual CYL with the PanOptix TFNT00 IOL

Preoperative refractive characteristics (both spherical and cylindrical errors) were similar in the three study groups (**Table 2**). Six months following the bilateral IOL-implantation residual refractions were measured. While the 71% of the eyes had a spherical equivalent refraction within 0.50 D from the target refraction, emmetropia in the case of the AT LISA tri lens, 97% of the eyes implanted with the Liberty IOL, and 82% of the eyes with the PanOptix lens achieved the same refractive outcomes (**Fig. 4a-c**). For the AT LISA tri, 93% of the eyes were within 1.00 D from emmetropia, while all eyes (100%) were within 1.00 D in the other two groups. **Table 3** presents the mean residual spherical (SPH) and cylindrical (CYL) refractions and the spherical equivalent refraction (SEQ) of the three groups. The Liberty IOL was found to be more efficient in spherical error correction than both the AT LISA tri and PanOptix IOLs (p=0.0091), hence, the postoperative SEQ of this particular study group was also lower in average (p=0.0025).

**Table 3:** Residual refractive errors measured six months postoperatively

	AT LISA tri 839MP	Liberty 677MY	PanOptix TFNT00	ANOVA p=	AT LISA tri vs. Liberty 677MY	AT LISA tri vs. PanOptix	Liberty 677MY vs. PanOptix
SPH (D)							
Mean ± SD	0.41 ± 0.56	0.07 ± 0.29	0.41 ± 0.47				
Range	-0.50; +1.75	-0.50; +0.75	-0.25; +1.25	0.0091*	0.0367*	>0.9999	0.0205*
CYL (D)							
Mean ± SD	-0.38 ± 0.34	-0.33 ± 0.40	-0.45 ± 0.37				
Range	-1.00; 0.00	-1.75; 0.00	-1.25; 0.00	0.3083	>0.9999	>0.9999	0.3884
SEQ (D)							
Mean ± SD	0.22 ± 0.52	-0.10 ± 0.27	0.19 ± 0.38				
Range	-0.75; +1.25	-0.50; +0.75	-0.50; +1.00	0.0025*	0.0083*	>0.9999	0.0110*
Axis (°)							
Mean ± SD	69.1 ± 65.0	50.0 ± 57.8	61.5 ± 61.3				
Range	0; 176	0; 173	0; 163	0.3646	0.5251	>0.9999	0.9670
**Notes:** * p values of 0.05 or less were considered to be statistically significant in all cases							

Residual astigmatism measured in the three groups is presented in **Fig. 4d-f**. The residual cylinder was within 0.50 D from emmetropia in 79% of the eyes implanted with the AT LISA tri lens, while 79% and 64% were within the same range in case of the Liberty and PanOptix IOLs, respectively. It should also be noted that if we focus on the eyes not further than 0.25 D cylinder from plano, the Liberty lens has the highest percentage of the eyes (68%), which is remarkably higher, than the 54% found in the AT LISA tri, and the 43% in the PanOptix groups.

***Evaluation of dysphotopsia***

**Fig. 5:**
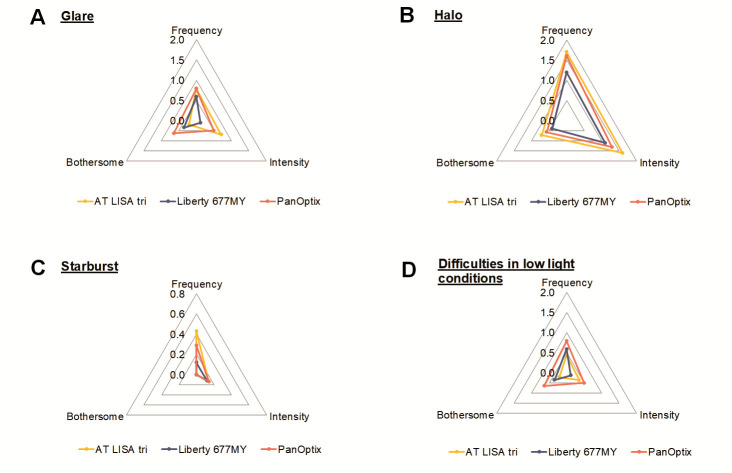
Evaluation of quality of vision in terms of the frequency, intensity and bothering feature of dysphotopic phenomena (glares, halos, starburst) and difficulties in low light condition. Data points represent the mean responses given on the 0 to 3 score scale of the McAlinden Quality of Vision questionnaire (0 represents the most favorable result; while 3 represents the least favorable result in each case). (A) Glare; (B) Halo; (C) Starburst; (D) Difficulties in low light conditions

**Fig. 6:**
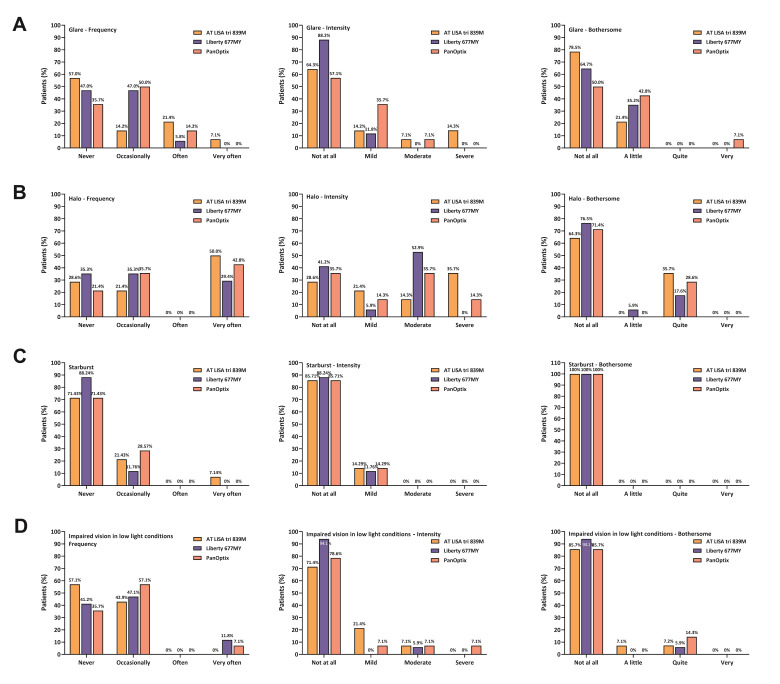
Detailed quality of vision evaluation in terms of the frequency, intensity and bothering feature of dysphotopic phenomena and difficulties in low light condition shows minor occurrence of dysphotopic events. Quality of vision was assessed with the McAlinden Quality of Vision questionnaire six months postoperatively. (A) Glare; (B) Halo; (C) Starburst; (D) Difficulties in low light conditions

Six months after the surgery, patients were tested for quality of vision, focusing on dysphotopic phenomena and other visual disturbances, using the McAlinden test [**11**]. **Table 4** and **Fig. 5** introduce the frequency, intensity and level of disturbances of the main visual disturbances, while **Fig. 6** shows the response categories and the distribution of responses given by the patients implanted with one of the three IOLs. Although ANOVA-tests could not reveal significant differences between the results obtained in the three groups (likely due to the low number of cases), it is obvious that the majority of patients offer favorable ratings in all groups and all cases (**Table 5**). Glare had the lowest prevalence rate in the AT LISA group (43.0%), while halo is the least frequent with the Liberty lens (64.7%) (**Table 4**). Also, starburst was much less frequent with the Liberty IOL (11.8%) than with the other two types of IOLs. AT LISA tri patients had the least difficulties in low light conditions (42.9%).

**Table 4:** Dysphotopic phenomena reported by the patients six months postoperatively. Frequency, intensity and the level of disturbance values were calculated based on McAlinden’s method.* Results are expressed as mean ± SD

	AT LISA tri 839MP	Liberty 677MY	PanOptix TFNT00	Significance (p)†
Glare				
Percentage of patients (%)	43.0	53.0	64.2	
Frequency	0.79 ± 1.10	0.59 ± 0.62	0.79 ± 0.70	0.7675
Intensity	0.71 ± 1.10	0.12 ± 0.33	0.50 ± 0.65	0.1228
Level of disturbance	0.21 ± 0.43	0.35 ± 0.49	0.64 ± 0.84	0.2522
Halo				
Percentage of patients (%)	71.4	64.7	78.6	
Frequency	1.70 ± 1.40	1.10 ± 0.99	1.30 ± 110	0.5552
Intensity	1.60 ± 1.30	0.41 ± 0.80	0.57 ± 0.94	0.5153
Level of disturbance	0.71 ± 0.99	0.12 ± 0.33	0.29 ± 0.47	0.6952
Starburst				
Percentage of patients (%)	28.6	11.8	28.6	
Frequency	0.43 ± 0.85	0.12 ± 0.33	0.29 ± 0.47	0.4115
Intensity	0.14 ± 0.36	0.12 ± 0.33	0.14 ± 0.36	0.9720
Level of disturbance	0.00	0.00	0.00	n.a.
Difficulties in low light conditions				
Percentage of patients (%)	42.9	58.8	64.3	
Frequency	0.43 ± 0.51	0.82 ± 0.95	0.79 ± 0.80	0.4000
Intensity	0.36 ± 0.63	0.06 ± 0.24	0.43 ± 0.94	0.2401
Level of disturbance	0.21 ± 0.58	0.12 ± 0.49	0.29 ± 0.73	0.7027
**Notes:** * 0, Never/ Not at all; 1, Occasionally/ Mild/ A little; 2, Quite often/ Moderate/ Quite; 3, Very often/ Severe/ Very				
† P values of 0.05 or less were considered to be statistically significant in all cases. ANOVA with the Kruskal-Wallis test was performed in all cases.				

Detailed analysis of the responses revealed that the frequency, intensity and bothersome of each phenomenon might not be parallel: e.g. in case of halos, 50.0% of AT LISA tri patients experienced halos very often, but only 35.7% of them found it bothersome (**Fig. 6b**). Similarly, more patients implanted with the Liberty IOL reported glare, than the AT LISA tri patients (53% vs. 43%), but 88.2% of them did not find it intense, compared to the AT LISA tri patients (64.3%) (**Fig. 6a**). The PanOptix lens received the least favorable ratings in most of the categories included in the questionnaire.

Visual disturbances occurred more often in scotopic conditions (evening, nighttime) than during daytime: 57.1% of PanOptix, 64.3% of AT LISA tri and 52.9% of Liberty patients had more difficulties in poor illumination than in well-lit conditions, while 21.4% (PanOptix), 7.1% (AT LISA tri) and 29.4% (Liberty) of the patients did not experience any difference between difficulties experienced in different light conditions. 

**Table 5:** The frequency, intensity and disturbing nature of dysphotopic phenomena with the three investigated multifocal IOLs six months postoperatively

	AT LISA tri (n=14)				Liberty 677MY (n=17)				PanOptix (n=14)				ANOVA p=	AT LISA tri vs. Liberty	AT LISA tri vs. PanOptix	Liberty vs. PanOptix
	Mean	SD	Min	Max	Mean	SD	Min	Max	Mean	SD	Min	Max				
Glare Frequency	0.79	1.10	0	3	0.59	0.62	0	2	0.79	0.70	0	2	0.7675	>0.9999	>0.9999	>0.9999
Glare Intensity	0.71	1.1	0	3	0.12	0.33	0	1	0.5	0.65	0	2	0.1228	0.2727	>0.9999	0.218
Glare Bothersome	0.21	0.43	0	1	0.35	0.49	0	1	0.64	0.84	0	3	0.2522	>0.9999	0.2931	>0.9999
Halo Frequency	1.70	1.40	0	3	1.2	1.3	0	3	1.6	1.3	0	3	0.5552	>0.9999	>0.9999	>0.9999
Halo Intensity	1.60	1.30	0	3	1.10	0.99	0	2	1.3	1.1	0	3	0.5153	0.7542	>0.9999	>0.9999
Halo Bothersome	0.71	0.99	0	2	0.41	0.8	0	2	0.57	0.94	0	2	0.6952	>0.9999	>0.9999	>0.9999
Starburst Frequency	0.43	0.85	0	3	0.12	0.33	0	1	0.29	0.47	0	1	0.4115	0.709	>0.9999	0.8405
Starburst Intensity	0.14	0.36	0	1	0.12	0.33	0	1	0.14	0.36	0	1	0.9720	>0.9999	>0.9999	>0.9999
Starburst Bothersome	0	0	0	0	0	0	0	0	0	0	0	0	n.a.	n.a.	n.a.	n.a.
Blurry Vision Frequency	0.29	0.47	0	1	0.59	0.8	0	3	0.64	0.5	0	1	0.1884	0.7716	0.2088	>0.9999
Blurry Vision Intensity	0.14	0.36	0	1	0	0	0	0	0.071	0.27	0	1	0.2909	0.3499	>0.9999	>0.9999
Blurry Vision Bothersome	0	0	0	0	0	0	0	0	0	0	0	0	n.a.	n.a.	n.a.	n.a.
Low Light Vision Frequency	0.43	0.51	0	1	0.82	0.95	0	3	0.79	0.8	0	3	0.4000	0.7617	0.6696	>0.9999
Low Light Vision Intensity	0.36	0.63	0	2	0.059	0.24	0	1	0.43	0.94	0	3	0.2401	0.3357	>0.9999	0.6590
Low Light Vision Bothersome	0.21	0.58	0	2	0.12	0.49	0	2	0.29	0.73	0	2	0.7027	>0.9999	>0.9999	>0.9999
When do you rather experience disturbances (0=daytime; 1= night-time; 2= both equally)	0.79	0.58	0	2	1.1	0.7	0	2	1	0.68	0	2	0.3746	0.4908	>0.9999	>0.9999
Distorted Vision Frequency	0.21	0.43	0	1	0.12	0.33	0	1	0.14	0.36	0	1	0.7565	>0.9999	>0.9999	>0.9999
Distorted Vision Intensity	0.29	0.61	0	2	0	0	0	0	0.29	0.83	0	3	0.1637	0.1985	>0.9999	0.6113
Distorted Vision Bothersome	0.14	0.36	0	1	0	0	0	0	0.29	0.83	0	3	0.2718	0.5371	>0.9999	0.4802
Double Vision Frequency	0.36	0.5	0	1	0.53	1	0	3	0.5	0.52	0	1	0.6592	>0.9999	>0.9999	>0.9999
Double Vision Intensity	0.71	1.1	0	3	0.53	1	0	3	0.79	1.1	0	3	0.5999	>0.9999	>0.9999	0.9363
Double Vision Bothersome	0.36	0.74	0	2	0.12	0.49	0	2	0.14	0.36	0	1	0.4541	0.6267	>0.9999	>0.9999
Fluctuation in Vision Frequency	0	0	0	0	0.35	1	0	3	0.43	0.51	0	1	0.0169	>0.9999	0.0157	0.1458
Fluctuation in Vision Intensity	0	0	0	0	0.24	0.75	0	3	0.14	0.36	0	1	0.3734	0.742	0.5984	>0.9999
Fluctuation in Vision Bothersome	0	0	0	0	0.059	0.24	0	1	0.071	0.27	0	1	0.6212	>0.9999	>0.9999	>0.9999
Focusing Frequency	0.36	0.5	0	1	0.24	0.75	0	3	0.21	0.43	0	1	0.3448	0.4387	>0.9999	>0.9999
Focusing Intensity	0.21	0.58	0	2	0.24	0.75	0	3	0.36	0.84	0	3	0.7729	>0.9999	>0.9999	>0.9999
Focusing Bothersome	0.21	0.8	0	3	0.059	0.24	0	1	0.14	0.36	0	1	0.7174	>0.9999	>0.9999	>0.9999
Distance-Depth perception Frequency	0	0	0	0	0.18	0.39	0	1	0.21	0.43	0	1	0.2070	0.4648	0.2973	>0.9999
Distance-Depth perception Intensity	0	0	0	0	0.18	0.39	0	1	0.14	0.36	0	1	0.2767	0.3718	0.7030	>0.9999
Distance-Depth perception Bothersome	0	0	0	0	0.18	0.73	0	3	0.071	0.27	0	1	0.6249	>0.9999	>0.9999	>0.9999
Spectacle use for near	0.43	0.65	0	2	0.41	0.62	0	2	0.5	0.65	0	2	0.9047	>0.9999	>0.9999	>0.9999

***Visual quality and patient satisfaction***

During the 6-months follow-up, all the patients were asked about their spectacle using habits. According to their responses, approximately two-thirds of AT LISA and Liberty patients (64.3% and 64.7%, respectively) did not need further visual correction for reading or other near visual tasks at all, while 42.9% of PanOptix patients needed to use spectacles for near vision (57.1% were spectacle independent).

**Fig. 7:**
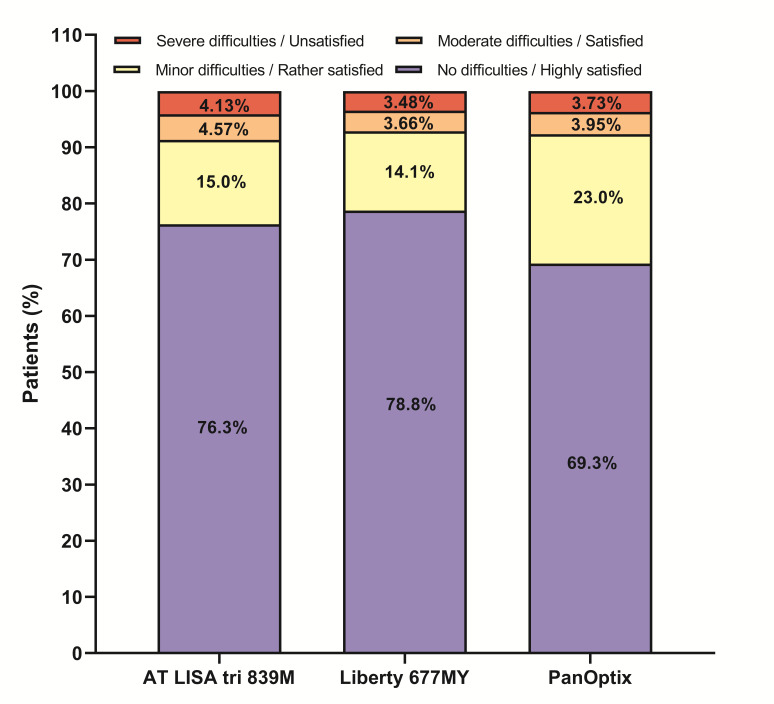
Most of the patients were highly satisfied, regardless of the trifocal intraocular lens implanted; however, Liberty patients reported the least vision-related difficulties

Taking all aspects into consideration, including their visual quality, occurrence of dysphotopic events and spectacle usage into account, patients were highly satisfied with the surgical and visual outcomes in all three groups (**Fig. 7**). In parallel with the visual acuity and refractive outcomes, the AT LISA tri and Liberty patients somewhat showed a higher satisfaction than those implanted with the PanOptix IOL.

## Discussion

The purpose of our current retrospective data collection and evaluation was to compare the refractive and visual outcomes six months following the binocular implantation of one of three MIOLs, all based on diffractive principles. Apart from visual acuity and autorefractometry measurements, a detailed analysis of visual function and dysphotopic artifacts was also performed. Pre- and postoperative data of the 90 eyes altogether (45 patients) were evaluated.

Uncorrected visual acuities measured at different distances were in accordance with the optical design of the lenses. Distance vision was shown to be of good quality in all three groups of patients, however, the Liberty lens was found to be the most efficient in distance vision correction. Intermediate vision reflected the additional power of each MIOLs: the AT LISA tri and the Liberty lenses provided superior visual acuities at 60 and 80 cm distances (with intermediate addition of 1.66 and 1.75 D, respectively), compared to the PanOptix lens. This is not surprising, knowing that the 2.17 D intermediate addition of the PanOptix model rather contributes to a 48 cm reading distance, which is closer to the near vision range. As we performed our intermediate visual acuity measurements at bigger distances, the “optimum” intermediate visual output of the PanOptix IOL might have remained undiscovered. Near vision assessment (measured at 30 and 40 cm) revealed that the AT LISA tri and Liberty lenses also perform better at presbyopia-correction. Although all three lenses have a similar near vision addition power (3.33 D for the AT LISA tri, 3.50 D for the Liberty and 3.25 D for the PanOptix), the AT LISA tri distributes 30%, the Liberty allocates 33% of light energy into the near focal point, while only 22% of the light is transferred to the near focus by the PanOptix lens. The visual outcomes observed also explained the spectacle using habits of the patients implanted with each MIOL-types: while approximately two-thirds of AT LISA tri and Liberty patients could achieve complete spectacle independence, only 57% of PanOptix patients could dispose of their glasses. The remaining 43% still required further near vision correction. More favorable spectacle using habits were published about the current investigated lenses by other authors, with more than 90% of the eyes achieving spectacle independence [**12**-**14**]. It must be remarked that in our current investigation, the toric models of the studied MIOLs could not be applied due to financial means of the subjects, therefore possible pre-existing corneal astigmatism was left uncorrected. This could have an adverse impact on visual outcomes, visual quality, and also on spectacle using habits [**15**-**17**]. 

The possible occurrence of dysphotopic disturbances is one of the main drawbacks of multifocal IOLs [**5**,**18**]. Due to the diffractive optical principles, these optical surfaces represent an increased risk for the higher incidence of unwanted photic phenomena [**19**,**20**]. The frequency of dysphotopic events was similar to those reported by others after the implantation of diffractive IOLs [**12**,**14**,**21**-**23**]. In general, all three investigated IOLs performed well and significant differences could not be revealed. This might be due to the moderate number of cases in each group; however, trends could be observed. Halo was shown to be the most frequent, intense and bothersome phenomenon, while starburst was reported only by a small minority of the patients (regardless of the lens they were implanted with), and none of them found it inconvenient. Glares were perceived with varying frequencies. However, Liberty patients suffered the least from this phenomenon and they were the least bothered in their daily activities. According to previous reports, it has been hypothesized that acrylic IOL materials with higher refractive index are more prone to cause photic disturbances, and a relatively flat anterior power curve and tall, square-edged optics are also supposed to contribute to the occurrence of positive dysphotopsia [**24**]. As for the IOLs in our current study, the AT LISA tri and the Liberty IOLs are made from a material that has a refractive index of 1.46. This is rather close to the 1.42 refractive index of the crystalline lens [**25**]. On the contrary, the PanOptix IOL has a refractive index of 1.55, one of the highest on the market. Another explanation for the higher degree of dysphotopsia in the case of the PanOptix lens might be the additional power of the optics. Kim et al. found that the subjects implanted with a lower add power of the same IOL model had greater satisfaction, more spectacle independence and fewer visual symptoms than those with a higher addition [**26**]. Their observations were recently confirmed by Altinkurt and Mulftuoglu, particularly in relation with the intensity of halos [**27**]. Our observations reflected their findings. The PanOptix lens has a near add similar to the other two MIOLs, but a higher intermediate addition (2.17 vs. 1.66 and 1.75 D, respectively). However, it has to be added that our sample of 15-17 patients in each studied IOL-groups was likely too low for drawing correct conclusions and we could not perform a detailed and precise correlation analysis between the IOL add powers and the presence of dysphotopsia.

An additional cause of dysphotopic events might be the energy distribution profile of the studied lenses. In case of a larger pupil (far vision) or poor light conditions, most of the light energy should be distributed to the far focal point and a smaller amount of light should arrive at the intermediate and near foci [**8**,**9**]. All the light landing in the untargeted foci will lead to visual disturbances, which often bother the patients and reduce their quality of vision [**2**-**4**,**6**,**7**,**9**]. In the case of the three lenses investigated in our current study, approximately 50% of the light energy contributed to the far focus (at 3.00 mm aperture diameter; therefore, with a mydriatic pupil, this ratio might be even bigger), therefore, energy distribution pattern was unlikely to be responsible for the slightly different levels of dysphotopsia reported by the patients. The energy loss of the three lenses were also similar (14.3, 11.0 and 12.0%). A major limitation of our study was that we were not able to collect consecutive data regarding the contrast sensitivity of the patients. This could have further enriched the picture we received about the visual quality of the patients. Based on the data, we could collect and analyze and we could support the hypothesis that the number of diffractive steps on the IOL surface might contribute to the realism of the retinal image, and hence, the overall quality of the patients’ vision. Of the three lenses, Liberty has the lowest number of diffractive rings (seven; while AT LISA tri has 21-29, and PanOptix has 15). Dysphotopsia evaluation revealed that the patients implanted with the Liberty lens might perceive dysphotopic events, but these are much less intense and disturbing according to their assessments. Additionally, light energy distribution also seemed to be optimal, as the Liberty lens provides high quality image at all distances. Hence, patients are more likely to achieve spectacle independence and are more satisfied with the overall surgical outcome.

The limitation of our study was that it was based on retrospective data collection and that only a six-month follow-up could be guaranteed for all cases. Based on the same principle as the current work, a prospective comparative cohort, extended with contrast sensitivity measurements and defocus curve comparisons, would be highly important to confirm our current findings and to serve as a much stronger base for any conclusions.

## Conclusion

Based on our results, we concluded that the three diffractive multifocal IOLs in our focus were all able to efficiently restore patients’ vision at all distances, however, unwanted photopic side effects could occur. The material and optical design of a MIOL does not only seem to be important for refractive correction and presbyopia-management, but also have a major impact on the postoperative quality of vision. Hence, a careful consideration should precede lens selection in each case.

**Acknowledgements**

None.

**Sources of Funding**

None.

**Disclosures**

None. 
